# Relationships between Individual and Social Resources, Anxiety and Depression in the Early Lockdown Stage by the COVID-19 in Chile

**DOI:** 10.3390/bs12100357

**Published:** 2022-09-25

**Authors:** Sergio Salgado, Carolina González-Suhr, Gabriela Nazar, Carlos-María Alcover, Raúl Ramírez-Vielma, Claudio Bustos

**Affiliations:** 1 Departamento de Administración y Economía, Universidad de La Frontera, Temuco 4811230, Chile; 2 Departamento de Psicología, Universidad de Concepción, Concepción 4070386, Chile; 3 Departamento de Psicología, Universidad Rey Juan Carlos, 28922 Madrid, Spain

**Keywords:** COVID-19, stress, anxiety, coping, social support, Chile

## Abstract

The coronavirus disease has exposed the population to psychosocial threats that could increase mental health problems. This research analyzed the relationships between emotional states (negative [−EWB] and positive [+EWB] experienced well-being), personal resources (resilient coping [RC]), dispositional resources (control beliefs about stress [BAS]), and social resources (social support [SS]), and anxiety and depressive symptoms in a sample of the Chilean population (*n* = 592), who answered an online questionnaire. Multiple and moderated multiple regression analyses were carried out. Depressive symptoms showed a positive relationship with −EWB (*β* = 0.805; *p* < 0.001) and negative relationship with +EWB (*β* = −0.312; *p* < 0.001), RC (*β* = −0.089; *p* < 0.01), BAS (*β* = −0.183; *p* < 0.001) and SS (*β* = −0.082; *p* < 0.001). Anxiety symptoms showed a positive relationship with −EWB (*β* = 0.568; *p* < 0.001), and a negative relationship with +EWB (*β* = −0.101; *p* < 0.03) and BAS (*β* = −0.092; *p* < 0.001). BAS moderated the relationship between experienced well-being and depression symptoms, and RC moderated the relationship between experienced well-being with both depression and anxiety symptoms. Findings confirm the buffering effect of personal and dispositional resources when facing a sanitary and social crisis. Moreover, they help to understand the role of internal psychological processes during a crisis and how to cope with life-threatening events.

## 1. Introduction

The coronavirus disease (COVID-19) is recognized as one of our time’s most significanthealth and humanitarian crises, registering more than 515 million confirmed cases and more than 6,240,000 deaths [1].

Different measures have been implemented as a form of control that have meant significant restrictions on daily life activities, work, and transportation, which has generated a significant vital disruption for many people. Uncertainty, changes in the work conditions, financial threats, and the loss of social support structure due to the constraint of mobility and contact expose the population to psychosocial risks, alter the daily experience of well-being, and increase vulnerability to mental health problems.

In this regard, early reports of studies carried out in countries initially affected by COVID agree on several negative psychological effects. As the pandemic spread to the west, different international [2] and local reports [3] have confirmed the impact of the pandemic on mental health, mainly anxiety and depression [4,5,6], but also feelings of loneliness [7], sleep disturbances [8], eating disorders [9], and substance abuse [10] among others. In Chile, where this research was carried out, a study performed on a representative sample of 13,648 Chilean people informed that 21.4% of the sample reported moderate or severe symptoms of anxiety and/or depression [3], and subsequent empirical studies have confirmed these results [11,12].

Previous research has shown that psychological well-being describes the experience of a stable, global, and deep state of well-being [13]. Thus, psychological well-being usually includes measures of the eudaimonic and hedonic aspects of well-being, with measures widely used in the scientific literature, such as positive and negative affectivity, concerning the hedonic elements [14]. However, experienced well-being has a narrower temporal referent since this construct “assesses momentary affective states and people’s feelings in real-time rather than relying on the memory of these states” [13], p. 2. The momentary affective states and people’s feelings in real-time can be an antecedent or even a precursor of mental health future states. These affective states are understood here as the experienced well-being, positive or negative [15], which can be considered as antecedents either improving or worsening mental health problems, such as anxiety or depressive disorders, specifically when they become persistent, disabling, or when they induce risk behaviors, such as drug or medication use. Since the experience of living in confinement was sudden and disruptive, it seems more relevant to measure immediately experienced well-being, i.e., that experienced daily, e.g., [16,17,18], hence the choice of a more momentary measure of affective states in this study.

As is well established in the scientific literature about stress, the relationship between psychosocial stressors and psychological and physical health is affected by the nature, number, and persistence of the contextual stressors, in interaction with the individual’s biological vulnerability, psychosocial resources, and learned patterns of stress coping [19]. Coping has long been considered a crucial process in the experience and treatment of multiple emotional and physical distress events [20]. Previous research has consolidated the growing evidence that positive emotions can broaden the range of possible coping strategies available to people during times and experiences of stress, thereby enhancing people’s resilience against present and future adversity [21,22]. Coping strategies influence emotional responses, and there is empirical evidence that active coping mechanisms, such as seeking active ways to solve problems or searching for help, are functional ways to deal with stress [23,24]. Resilient coping can allow people to overcome adversity from a highly stressful psychosocial event [25]. In addition, clinical researchers have also demonstrated that individual resilience can moderate the impact of stress on anxiety and depressive symptoms [22]. Recent scientific studies in a population affected by COVID-19 indicate that stress experienced is related to coping strategies [26] and that the use of coping strategies was associated with decreased anxiety and depression during the COVID-19 confinement and pandemic experiences [27,28].

Cognitive and subjective notions people have about stress, and the beliefs as to what extent control can be exerted over it [29], have been considered in scientific research as resources to deal with this experience, which in turn lead to health outcomes, particularly well-being and mental health [30]. Stress beliefs are “a form of lay belief or theory about stress held by an individual” [31], p. 595; [32]. These beliefs can be built on both theory and scientific evidence, as well as past experiences concerning experiential and vicarious situations [31]. Moreover, stress beliefs can be situation-specific or generalized [29]. Previous empirical research has found that negative stress beliefs moderate the affective response to real-life stressors [29]. Thus, when people experienced social stress, those with high negative beliefs about stress were more prone to daily high negative affect; conversely, this association was lower in those with low negative beliefs about stress. In addition, individuals who believe that stress is controllable show greater positive affect throughout daily experiences [29]. In short, the accumulated research evidence in the stress domain [33,34] suggests that both situation-specific and generalized stress beliefs could be a promising new construct in explaining health and identifying their influences on short- and long-term health.

Among other resources to face stressful life events, there is a broad range of evidence sustaining the effects of social support on well-being [35] and its buffering effect on the psychological consequences of traumatic experiences, such as pandemics and natural disasters [36,37]. Over the past decades, scientific research has found that the main evidence for the buffering model of social support (i.e., “the process of support protecting persons from potentially adverse effects of stressful events” [38] p. 310 is associated with when such social support refers to the perceived availability of interpersonal resources that enable individuals to respond to the demands caused by stressful life events [38,39]. Social support constitutes an important pool of resources provided by other people that accumulated into personal resources [40]. Thus, empirical evidence shows that social support may be a central element of health and well-being as, together with personal resources, it is related to the overall sense of identity [40]. This follows as it relates self-concept to social network and interpersonal relationships [38]. In particular, the buffering effect of social support as a resource has been found in coping with high-stress situations, such as disasters and highly disruptive life events [41,42]. For instance, Fluharty et al. (2021), in a study carried out during the first 21 weeks of the COVID-19 lockdown, found that although mental health symptoms decreased over time for all coping strategies, only socially-supportive coping was associated with an effective and faster decrease in anxiety and depressive symptoms [43]. These research results indicate a potential protective effect of social support on psychological distress associated with the lockdown experience.

In sum, to understand how the COVID-19 crisis could affect people’s mental health, we take Hobfoll’s well-established Conservation of Resources Theory (COR) [44,45] as a framework. This theory considers the context and the individual responses to these circumstances, which can lead to a decrease in mental health due to the loss of valuable resources (affective, personal, social). Specifically, this study set out to analyze the relationships between emotional states (experienced well-being, negative [−EWB] and positive [+EWB]), personal resources (resilient coping [RC]), dispositional resources (beliefs about stress [BAS]), and social resources (social support [SS]) and anxiety and depressive symptoms as mental health consequences of the COVID-19 pandemic in a sample of the Chilean population.

**Figure 1:**
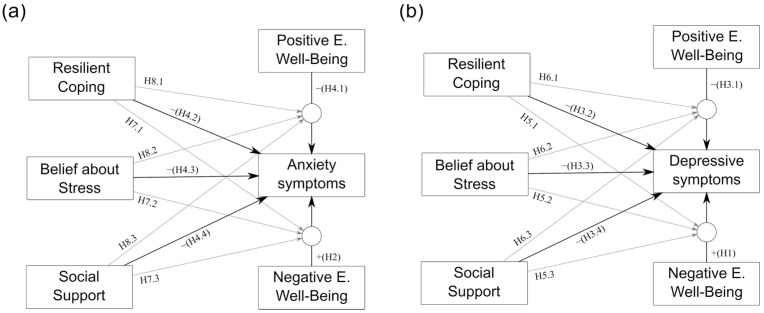
Model of proposed hypotheses for (**a**) anxiety and (**b**) depression.

Based on the above theoretical models and empirical evidence, we proposed the following hypotheses: There is a positive relationship between −EWB and depressive (H1) and anxiety (H2) symptoms; and a negative relationship between +EWB, RC, BAS, and SS with depression (H3.1, H3.2, H3.3, and H3.4, respectively) and anxiety symptoms (H4.1, H4.2, H4.3, and H4.4, respectively). Additionally, we expect that RC, BAS, and SS, have a moderating effect on the relationship between −EWB and depression (H5.1, H5.2, and H5.3, respectively) and +EWB and depression (H6.1, H6.2, and H6.3, respectively); and, that RC, BAS, and SS have a moderating effect on the relationship between −EWB and anxiety symptoms (H7.1, H7.2, and H7.3, respectively) and +EWB and anxiety symptoms (H8.1, H8.2, and H8.3, respectively). Figure 1a,b.

## 2. Materials and Methods

### 2.1. Research Design and Participants

According to the purpose of the study, a cross-sectional correlational study was conducted on a non-probabilistic sample aimed at a general Chilean adult population. The sample consisted of 591 participants who took part in the study (592 responses to the online questionnaire were obtained, of which a duplicate case identified through e-mail was eliminated), with an average age of 37.63 years (*SD* = 12.85). 76% were female (*n* = 449), 23.7% were male (*n* = 140), and 0.3% identified as another gender (*n* = 2). All the participants lived in Chile. Most of the participants had a full-time day job (*n* = 269; 45.5%), but they also had part-time jobs (*n* = 43; 7.3%), were independent workers (*n* = 82; 13.9%), students (*n* = 79; 13.4%), unemployed (*n* = 55; 9.3%), retired (*n* = 30; 5.1%), homemakers (*n* = 24; 4.1%) and others (*n* = 9; 1.5%). 37.5% (*n* = 222) reported having postgraduate studies, 43.3% (*n* = 256) graduate studies, 8.3% (*n* = 49) technical studies, 10.7% (*n* = 63) had high school and 0.2% (*n* = 1) elementary school.

### 2.2. Instruments

Experienced well-being was measured by the subscale named alike from The Pemberton Happiness Index scale [13]. It is a 10-item measure, five items of +EWB (e.g., ‘I did something fun with someone’), and five of −EWB (e.g., ‘I was worried about personal matters’). Response options were: ‘Yes, it happened to me last days’ or ‘No, it did not happen to me last days’. Internal consistency, as measured by Cronbach’s Alpha, was above 0.89, and the results from this initial validation study provided very good support for the scale’s psychometric properties (single-factor structure, and convergent and incremental validity) [13]. In this study, Cronbach’s alpha was 0.73 for +EWB and 0.78 for −EWB.

Resilient Coping was measured by the Spanish version of the Brief Resilience Scale (BRS) [25,46], a four-item scale: ‘I look for creative ways to alter difficult situations’. The format is a Likert response with 5 anchor points, from 0 = does not describe me at all to 6 = describes me very well. Internal consistency as measured by Cronbach’s Alpha was 0.86. The scale demonstrated good psychometric properties in terms of criterion validity, homogeneity indices, and dimensional structure [25]. In this study, Cronbach’s alpha was 0.83.

Beliefs about stress were measured with the Beliefs About Stress Scale (BASS) [31], subscale Control Beliefs, composed of three items: ‘Stress is something I can control to some extent’. The answering format was a 4-point Likert scale: 1 completely disagree to 4: completely agree. The scales showed good to acceptable internal consistency (Cronbach’s Alpha 0.73–0.87) and retest-reliability (*r*_tt6-8_ 0.61–0.81). Correlations with optimism, pessimism, neuroticism, and somatosensory amplification (*r* 0.15–0.47) indicated high to medium discriminant validity. Moreover, stress beliefs appear to be multidimensional and stable over time [34]. In this study, Cronbach’s alpha was 0.86.

Social support was measured with the abbreviated version of the Medical Outcome Study Social Support Survey (MOS-SSS) [47,48] composed of four items, scored on a 5-point response from 1 = never to 5 = always, sample item is (e.g., ‘Do you have someone who helps you to solve your personal problems?’). The reliability of this 4-item scale was acceptable (Cronbach’s Alpha was 0.83). In addition, a good fit was obtained in terms of its factorial structure [47]. In this study, Cronbach’s alpha was 0.80.

Anxiety was measured by the Generalized Anxiety Disorder-7 (GAD-7) [49] in its Spanish adaptation from [50], which consists of seven items about people‘s experiences during the last week. An example item statement is ‘Feeling nervous, anxious or on edge thinking’ and the participant must respond with one of the four alternatives from 0 = never to 3 = almost every day. Cronbach’s Alpha reached an excellent value (0.93). The scale was shown to be one-dimensional through factor analysis (explained variance = 72%). Likewise, the scale obtained good indices in terms of inter-rater validity, criterion validity, and discriminant validity [50]. In this study, Cronbach’s alpha was 0.90.

Depression was measured by the Patient Health Questionnaire Depression Scale (PHQ-9) [51] adapted to Spanish, consisting of nine items about the frequency of a personal situation in the last week, scored on a 4-point Likert response format from 0 = never to 3 = almost every day. An example item statement is: ‘Little interest or pleasure in doing things’ [52]. The internal reliability of the PHQ-9 was high, with a Cronbach’s Alpha of 0.89. Construct validity was established by the strong association between PHQ-9 scores and functional status, disability days, and symptom-related difficulty [51]. In this study, Cronbach’s alpha was 0.90.

Both scales GAD-7 and PHQ-9 are used as screening tools in clinical contexts, however, they are also widely used for research purposes [49,50,51].

### 2.3. Procedure and Ethics

On 16 March 2020, the Chilean Government began to decree social distancing and confinement measures. They progressively increased restrictions on productive, commercial, and consumption activities as different regions detected contagions and deaths. The data were collected via an online questionnaire (Questionpro) between 24 March and 23 April. The recruitment strategy combined professional networks, social networks, professional associations, undergraduate and graduate students, and personal contacts, who were invited to participate and disseminate this invitation among their network of contacts (snowball strategy). The participants took an average of 27 min to complete all the instruments. When necessary, they could save their progress and take it up again when convenient.

First, the psychometric properties of the instruments were analyzed. Afterward, a multiple regression analysis was performed to test the relationship between the independent variables (+EWB and −EWB, RC, BAS, and SS) and the two dependent variables (depression and anxiety symptoms).

Then, a moderated multiple regression analysis was carried out to explore the potential moderating role of RC, BAS, and SS in the relationship between +EWB and −EWB and depression and anxiety symptoms. These analyses included age, gender (female = 1; male = 2; other = 3), and educational level (elementary school = 1; high school = 2; technical studies = 3; University degree = 4; Postgraduate = 5) as control variables.

To probe the moderation, two techniques were used. First, the pick-a-point approach was used, plotting the simple regression lines for three values of the moderating variable: mean, −1 sd, and +1 sd. Second, we use the J-N technique, which indicates over what range of the moderator the effect of the predictor is significant or not significant.

The project underwent assessment by the Ethics and Bioethics Committee of the Universidad de Concepción (CEBB 650-2020, March 2020), and the participants were asked to sign an informed consent. When the proposed conditions were agreed upon, the participants marked a square indicating they were over 18 years, had been informed, and understood the nature of this study and their decision to participate voluntarily.

## 3. Results

### 3.1. Psychometric Properties of the Instruments

A measurement model was tested using CFA, in which all variables with all their items were considered to verify that the scales had convergent validity (relationships between items and their factors) and discriminant validity (that the items are not strongly related to other factors, and that the factors do not have high correlations with each other). The CFA showed a good fit of the data to the model (*x2/df* = 2.033; *CFI* = 0.964; *TLI* = 0.961; *SRMR* = 0.06 y *RMSEA* = 0.042 [0.038, 0.045]), which also supports discriminant validity of the measures. Table 1 shows the descriptive values, internal consistency (polychoric), and Pearson correlations.

The model for depression symptoms as a dependent variable presented a slight increase in the variance of the residuals when the predicted value increased. In contrast, the model for anxiety as a dependent was homoscedastic, observing in a first analysis that the residuals had positive skewness, controlled using the logarithm of anxiety. The moderation models meet the assumptions of linearity and normality of residuals.

### 3.2. Multiple Regression Analysis

Regarding the dependent variable depression, the regression model explained 47.6% of the variance (*F* (12, 578) = 45.77, *p* < 0.001, adjusted *R^2^* = 0.476).

In line with the hypotheses H1, H3.1, H3.2, H3.3, and H3.4 (see Table 2, non-moderated) a positive significant coefficient was obtained for the −EWB and significant negative coefficients for +EWB, RC, BAS, and SS. No control variables showed a significant relationship with depression symptoms.

Regarding anxiety, the regression model explained 37.9% of the variance (*F* (12, 578) = 31.09, *p* < 0.001, adjusted *R^2^* = 0.379). In line with the hypotheses H2, H4.1, and H4.4 (see Table 3, non-moderated), significant weights in a positive direction were shown with −EWB, and in a negative direction with +EWB and BAS. The control variable gender showed a significant regression weight, which indicated that men showed a lower level of anxiety compared to women. Hypotheses H4.2 and H4.3 were not supported.

### 3.3. Moderated Multiple Regression Analysis

Subsequently, a moderated multiple regression analysis was carried out to explore the potential moderating role of RC, BAS, and SS in the relationship between +EWB and −EWB, and depressive and anxious symptoms. Regarding depression symptoms, when comparing the moderated regression model with the original (non-moderated) model, a significant difference was observed that supports the significance of the moderation (*F* (6, 572) = 5.318, *p* < 0.001).

**Figure 2:**
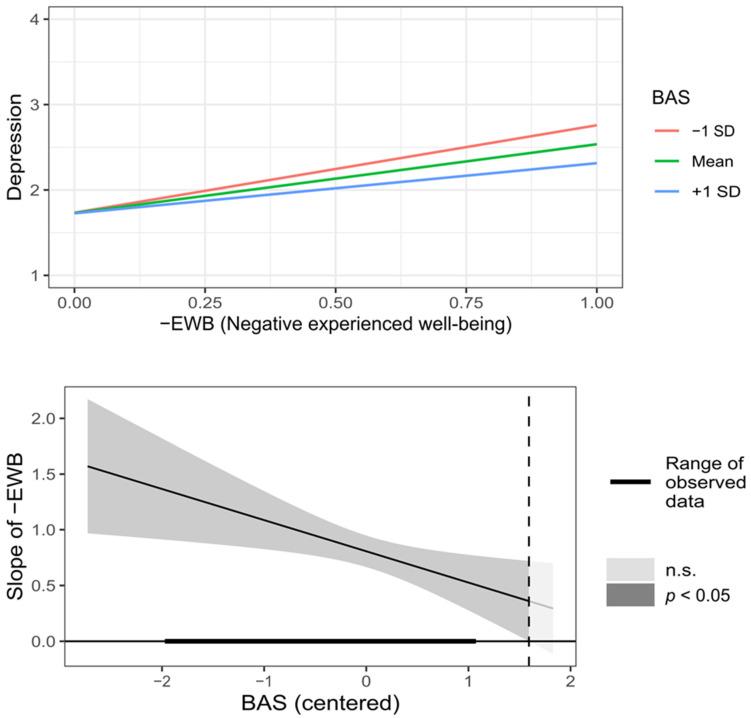
Graphic representation of BAS (Beliefs about stress) moderation on the relationship between −EWB (Negative experienced well-being) and depression symptoms.

**Figure 3:**
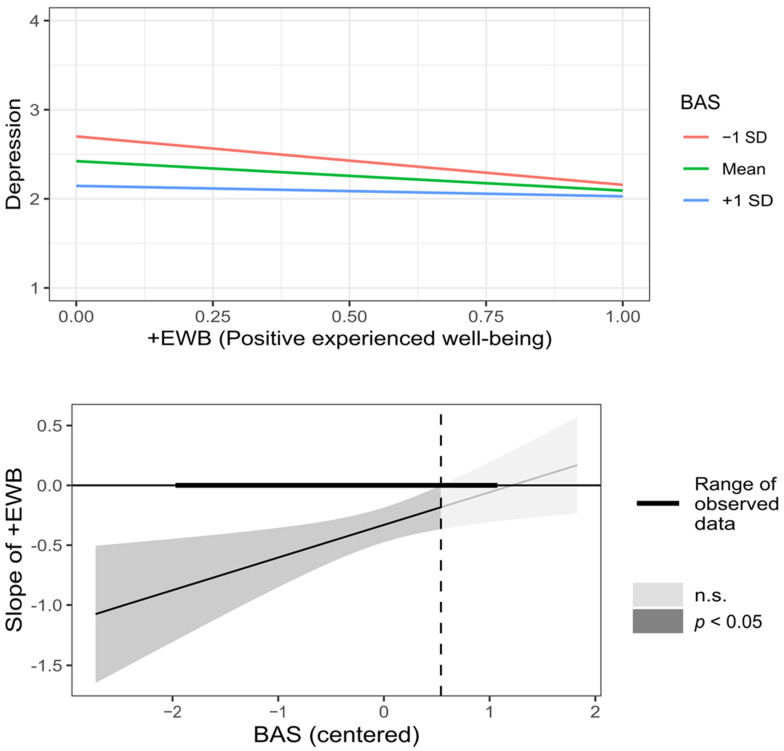
Graphic representation of BAS (Beliefs about stress) moderation on the relationship between +EWB (Positive experienced well-being) and depression symptoms.

**Figure 4:**
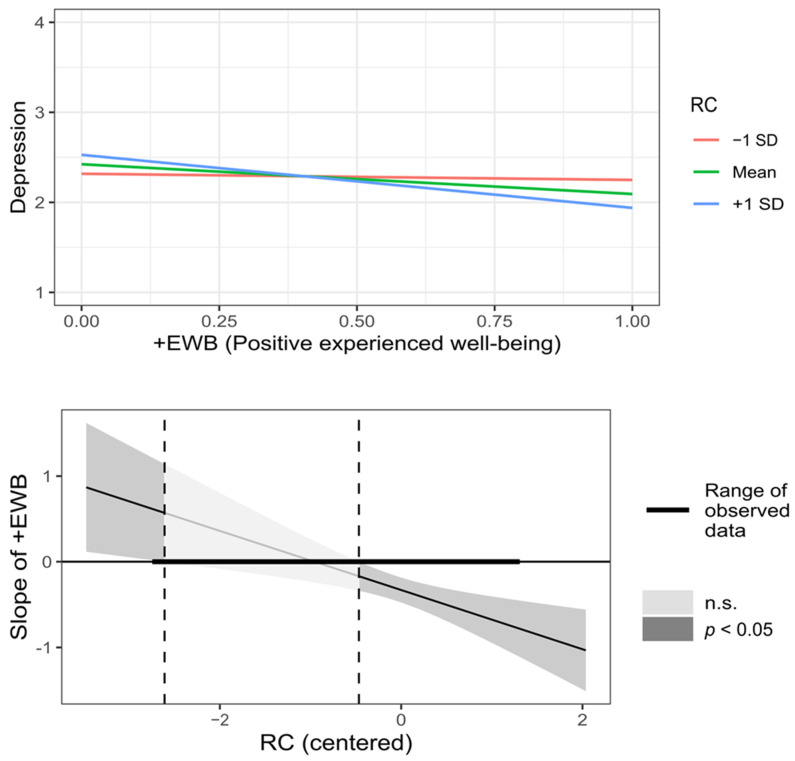
Graphic representation of BC (Resilient coping) moderation on the relationship between +EWB (Positive experienced well-being) and depression symptoms.

As shown in Table 2 (moderated), BAS significantly moderated the relationship between −EWB and depression symptoms. In this case, the relationship was positive for all BAS values, so as control beliefs increased, decreased the relationship between −EWB and depression symptoms (see Figure 2). On the other hand, BAS and RC significantly moderated the relationship between +EWB and depression symptoms. Here, for all BAS values, the relationship between +EWB and depression symptoms was inverse; that is, as BAS increased, the strength of that relationship decreased (see Figure 3). Additionally, when RC was low (less than one SD), the effect of +EWB on depression symptoms was near zero; as RC increased, the reverse relationship became progressively stronger (see Figure 4). The moderating effect on the relationship between −EWB and +EWB, and depression symptoms, support the hypotheses H5.2, H6.1, and H6.2, whereas hypotheses H5.1, H5.3, and H6.3 were not supported.

**Figure 5:**
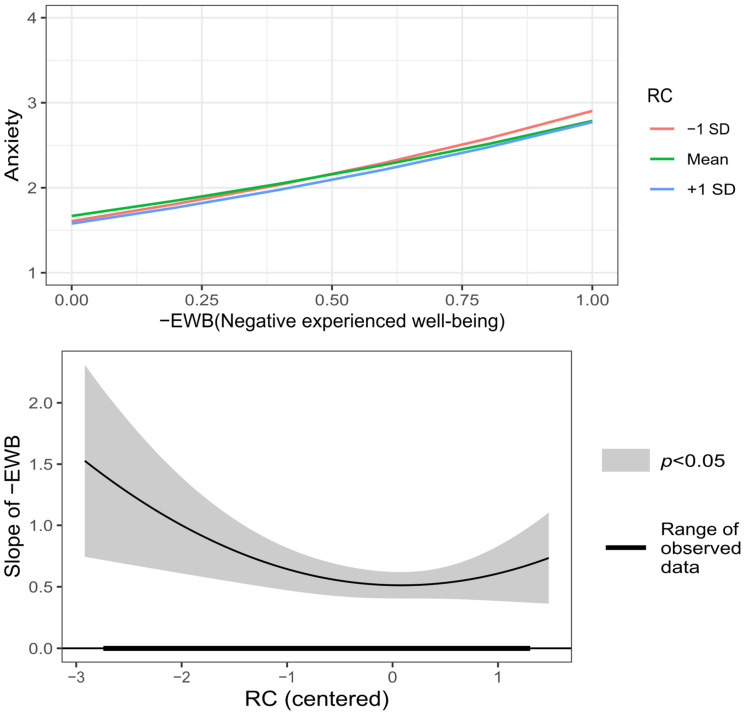
Graphic representation of RC (Resilient coping) moderation on the relationship between −EWB (Negative experienced well-being) and anxiety symptoms.

**Figure 6:**
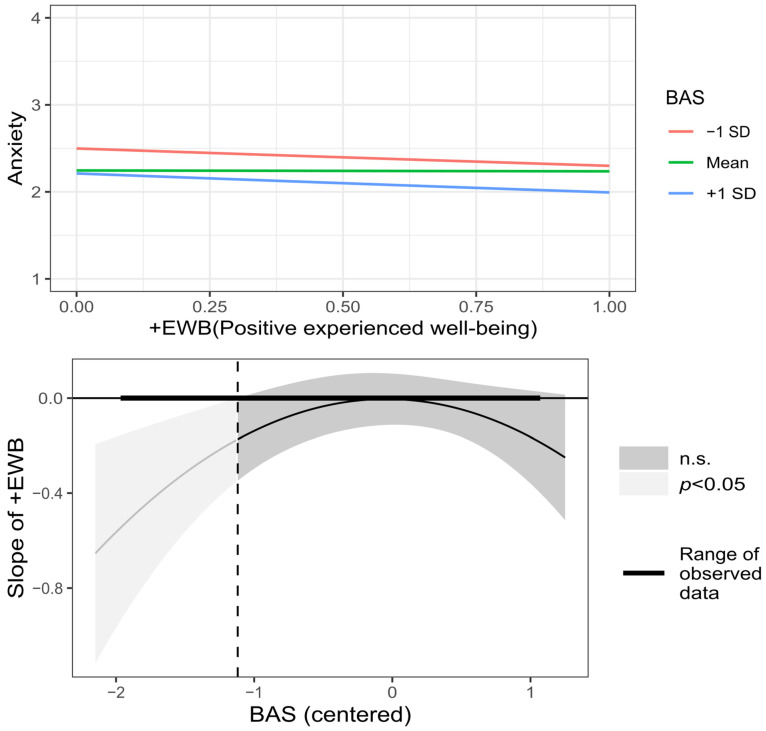
Graphic representation of BAS (Beliefs about stress) moderation on the relationship between +EWB (Positive experienced well-being) and anxiety symptoms.

Regarding the moderation of the relationship between +EWB and −EWB, and anxiety, when comparing the linear moderated regression model with the original model, there was no significant difference (*F* (6, 572) = 1.018, *p* = 0.41). Then, it was taken into account that the graphic exploration of these effects revealed that the moderation of BAS and RC was not linear: A distinctive U-shaped curve was detected in the effect of RC in the relation between −EWB on anxiety, and an inverted U-shaped curve in the effect of BAS in the relation between +EWB on anxiety. Therefore, the polynomial in degree two (quadratic) for BAS and RC was added to the moderated regression model, and this model did show a significant change compared to the model with linear moderation (*F* (6, 566) = 3.277, *p* = 0.003). As shown in Figure 5, for all viable RC values, the direct relationship between −EWB and anxiety is significant. When RC decreases below the mean, the relation between −EWB and anxiety becomes stronger, but the relation between −EWB and anxiety is nearly the same for values of RC near and higher than the mean. In the case of BAS, as shown in Figure 6, the region of no significance starts at 1.83 and ends at 4.39; so, for BAS lower than 1.83 (8.12% of the sample), there is a significant inverse relation between +EWB and anxiety; between 1.83 and the mean of BAS (2.94), the relation tends to zero and for values higher than the mean, the relation becomes stronger, but not enough to be outside the region of no significance. Therefore, regarding the moderating effect on the relationship between −EWB and +EWB, and anxiety, the hypotheses H7.1 and H8.2 were supported, and the hypotheses H7.2, H7.3, H8.1, and H8.3 were not supported.

## 4. Discussion

Following the conservation of resources theory (COR) [44,45], the purpose of this work was to test the relationships between emotional states (experienced well-being, negative [−EWB] and positive [+EWB]), personal resources (i.e., resilient coping [RC]), dispositional resources (i.e., beliefs about stress [BAS]), social resources (i.e., social support [SS]), and anxiety and depressive symptoms as mental health consequences of the COVID-19 pandemic in a sample of the Chilean population. According to COR theory, lower levels of personal, social, and material resources are important determinants of poorer psychological adaptation to adverse situations. Specifically, the COR theory postulates that the loss of resources not only is more powerful than resource gain in magnitude, but also it tends to affect people more quickly and at an increasing rate over time, which is relevant in the context of the COVID -19 crisis.

Meta-analytic results on the prevalence of depression in the general population during the COVID -19 outbreak [53] show that the pooled prevalence of depression was 25% (95% CI: 18–33%), which appears to be seven times higher than the global estimated prevalence of depression in the general population in 2017 (3.44%) [54]. Additionally, in previous empirical studies, it has been found that during the initial stages of lockdown, Chile showed high levels of stress, which is expected in a country with a low level of social protection, thus the approach of this study seems particularly relevant [55,56]. These results, showing increased levels of stress, anxiety, and depressive symptoms, have been confirmed in both cross-sectional and longitudinal studies with data obtained during the first year of the pandemic and even in subsequent years, e.g., [53,57,58,59]. Moreover, these empirical results are confirmed cross-culturally, as empirical evidence shows similar indicators of worse mental health across geographic areas, e.g., [60,61,62,63].

Our findings revealed that variables related to personal resources (coping strategies), dispositional resources (beliefs about stress) and affective states (experienced positive well-being), and social resources (social support) are negatively related to depression symptoms in confinement, whereas experienced negative well-being is positively related. Our results allow a first approximation to the identification of the factors associated with the presence of depressive symptomatology in lockdown, such as EWB, and add RC, BAS, and SS, to other personal and social factors inversely related to depression in confinement, such as group membership, personal identity strength, and perceived personal control [64], or psychological flexibility [65].

Regarding anxiety, results show a negative relationship between control belief of stress and positive well-being, and positive with negative well-being. Once again, our results not only note the increased anxiety due to confinement, a general phenomenon across countries during the pandemic [36,66,67] and a proven increase in comparison with pre-pandemic anxiety levels (e.g., [68]) but identify possible associated factors that mitigate its incidence, adding into the knowledge of other effective coping behaviors [27].

Furthermore, our study has found interesting differences in the moderating effects of personal, dispositional, and social resources in the relationship between dominant affective states and depressive and anxiety symptomatology. Thus, while the effects are linear for +EWB and RC in the relationship between +EWB and depression symptoms, and for BAS in the relationship between −EWB and depression symptoms, that moderating effect of both variables is quadratic for the relationships between +EWB and anxiety (but just BAS was significant).

Contrary to expectations, we did not find any direct or moderating effect of SS on the study variables. Although this contradicts much of the research carried out during the COVID-19 pandemic [26,64,65,66,67,68,69], there is also evidence that government-imposed stay-at-home and personal distancing were independently associated with higher symptoms of depression and generalized anxiety disorder, beyond the potential protective effects of available social resources, such as SS and social network size [55]. This may be as a result of isolation and social distance being imposed suddenly and our data came from the first weeks of confinement, all of which could have limited the relations and communication with social networks and perceived SS. Future longitudinal studies should check whether, in subsequent weeks, the role of SS was maintained or altered, and if so, in which direction.

The results obtained allow us to go beyond the mere confirmation of the worsening of mental health during confinement and represent an additional step in identifying factors that can suffer the consequences of current and future lockdowns and severe confinements (for instance, currently in several regions of China). This practical value of our study will have to be corroborated by data from longitudinal designs that allow confirmation of the possible existence of causal relationships between variables, as well as the moderation effects detected.

### 4.1. Strengths and Limitations

Although time has passed since these data were collected, the results obtained are timely and relevant for various reasons. First, they show that some psychological variables do not develop linear effects, which is commonly reported in the literature, so their scientific novelty does not rely solely on the situation caused by the COVID-19 pandemic. Second, we believe that the initial reaction of people in a situation of abrupt limitation of social routines, as it was at early lockdown, is not exhausted in this event, and it can be extrapolated to other hard and inadvertent situations. These findings help us to better understand internal psychological processes during a crisis and to be prepared for other public health crises, natural disasters, or armed conflicts, that profoundly affect our lives.

As a consequence of the sampling process, based on the snowball technique, the participants are a non-probabilistic sample of the population. Future studies in this research domain should include concentrating on specific people or contexts (professional groups, vulnerable groups, youth, or older people among others) or representative samples to identify more accurately the differential effects in each setting. Although the sample is unbalanced in terms of gender, it was possible to analyze its effect, concluding what is already known in the literature regarding a higher prevalence of anxiety for females. Additionally, the cross-sectional nature of the design only allows the identification of associations between variables, and future research could be based on experimental, quasi-experimental, or longitudinal designs. In any case, the relationships found are largely supported, albeit in other settings, by previous literature. However, respondents were not tested before for mental or physical disease, and mental and physical chronic diseases were not considered for the present research. In addition, some other sociodemographic variables or family/work/financial data that have been shown to be relevant to people’s mental and physical lives during the pandemic outbreak were not considered and should be included in future studies, using other techniques such as diary data collection or in-depth interviews.

### 4.2. Practical Implications

The results obtained allow us to go beyond the mere confirmation of the worsening of mental health during confinement and represent an additional step in identifying factors that can suffer the consequences of current and future lockdowns and severe confinements (for instance, currently in several regions of China). This practical value of our study will have to be corroborated by data from longitudinal designs that allow confirmation of the possible existence of causal relationships between variables, as well as the moderation effects detected. Additionally, some other practical recommendations arise. First, to improve personal resources conservation, it is important to promote the ability to focus on positive experiences and intensify and prolong these positive feelings. Resilience training and self-esteem development in educational curricula, together with pedagogical practices such as systematic positive feedback, can be strategies to enhance personal resources. Second, in terms of social support and protection, it is relevant to use social networks and new digital technologies to train and reinforce social resources that favor personal growth and mutual care.

## 5. Conclusions

The coronavirus disease (COVID-19) has disrupted the daily experience and exposed the population to psychosocial threats that alter well-being and increase vulnerability to mental health problems. These findings support the relationship between affective states and immediate emotional experience, namely experienced well-being, with depressive and anxiety symptoms during confinement. Additionally, it highlights the protective role of personal and social resources. Specifically, someone’s resilience, control beliefs of stress, and social support were found as relevant assets with a buffer effect that can impede that experiences of distress under a crisis turned into mental health problems.

## Figures and Tables

**Table 1:** Mean, standard deviation, internal consistency, and Pearson’s correlations for the variables under study.

Variable	*n*	Mean	*SD*	αord	Pearson’s Correlations						
					(1)	(2)	(3)	(4)	(5)	(6)	(7)
1. Depression symptoms	591	1.846	0.6377	0.9042	1	0.72 **	−0.23 **	−0.48 **	−0.43 **	−0.33 **	0.53 **
2. Anxiety	591	2.106	0.7244	0.9041		1	−0.12 **	−0.40 **	−0.33 **	−0.26 **	0.52 **
3. SS	591	3.752	0.9038	0.8044			1	0.12 **	0.18 **	0.21 **	−0.10 *
4. BAS	591	2.949	0.7788	0.8682				1	0.56 **	0.28 **	−0.29 **
5. RC	591	3.719	0.7557	0.8369					1	0.32 **	−0.27 **
6. +EWB	591	0.6968	0.2799	0.7381						1	−0.17 **
7. −EWB	591	0.5739	0.2849	0.7866							1

Note: αor: Cronbach’s alpha based on polychoric correlation. SS: Social Support; BAS: Beliefs about stress; REC: Resilient Coping; +EW: Positive experienced well-being; −EW: Negative Experienced well-being. * *p* < 0.05; ** *p* < 0.01.

**Table 2:** Non-moderated and Moderated regression analysis summary for the interaction between SS (Social Support), BAS (Beliefs about stress), and RC (Resilient Coping); and +EWB (Positive experienced well-being), and −EWB (Negative experienced well-being) predicting depression symptoms.

Variable	Non-Moderated		Moderated	
	*Β*	CI 95%	*Β*	CI 95%
(Intercept)	3.281 **	[2.331, 4.231]	1.944 **	[0.769, 3.119]
SS	−0.082 **	[−0.126, −0.038]	−0.025	[−0.177, 0.126]
BAS	−0.183 **	[−0.243, −0.123]	−0.194	[−0.393, 0.003]
RC	−0.089 **	[−0.152, −0.028]	0.219 *	[0.006, 0.434]
+EWB	−0.312 **	[−0.460, −0.165]	0.158	[−0.594, 0.910]
−EWB	0.805 **	[0.661, 0.949]	2.463 **	[1.669, 3.257]
Gender = Male	−0.069	[−0.159, 0.019]	−0.048	[−0.135, 0.040]
Gender = Other	0.317	[−0.329, 0.963]	0.463	[−0.172, 1.098]
Education = High School	−0.100	[−1.017, 0.817]	−0.097	[−0.997, 0.802]
Education = Technical studies	−0.303	[−1.225, 0.620]	−0.341	[−1.246, 0.563]
Education = University Degree	−0.334	[−1.248, 0.580]	−0.337	[−1.233, 0.559]
Education = Postgraduate	−0.470	[−1.385, 0.445]	−0.462	[−1.359, 0.434]
Age(years)	−0.003	[−0.007, 0.000]	−0.003 *	[−0.007, −0.000]
SS x +EWB			−0.003	[−0.153, 0.148]
SS x −EWB			−0.083	[−0.229, 0.064]
BAS x +EWB			0.273 **	[0.070, 0.476]
BAS x −EWB			−0.280 **	[−0.492, −0.068]
RC x +EWB			−0.345 **	[−0.561, −0.129]
RC x −EWB			−0.140	[−0.359, 0.078]
Adjusted *R^2^*	0.476		0.514	
*F*	45.766 **		33.651 **	
Δ*R^2^*			0.027	
Δ*F*			5.318 **	

Note: * *p* < 0.05; ** *p* < 0.01. All numeric predictor variables are centered to the mean.

**Table 3:** Non-moderated and Moderated analysis summary for SS (Social Support), BAS (Beliefs about stress), RC (Resilient Coping), +EWB (Positive experienced well-being), and −EWB (Negative experienced well-being) predicting anxiety symptoms.

Variable	Non-Moderated		Moderated	
	*Β*	CI 95%	*Β*	CI 95%
(Intercept)	0.904 **	[0.342, 1.465]	0.239	[−0.847, 1.325]
SS	0.002	[−0.024, 0.028]	0.051	[−0.043, 0.144]
BAS	−0.092 **	[−0.128, −0.057]	−0.555	[−1.188, 0.079]
BAS2			0.082	[−0.030, 0.194]
RC	−0.027	[−0.064, 0.010]	0.44	[−0.225, 1.105]
RC2			−0.049	[−0.147, 0.049]
+EWB	−0.101 *	[−0.189, −0.015]	−1.207 *	[−2.307, −0.107]
−EWB	0.568 **	[0.484, 0.654]	2.167 **	[1.069, 3.264]
Gender = Male	−0.081 **	[−0.133, −0.028]	−0.082 **	[−0.135, −0.029]
Gender = Other	0.065	[−0.317, 0.447]	0.085	[−0.295, 0.466]
Education = High School	−0.106	[−0.648, 0.436]	−0.037	[−0.574, 0.501]
Education = Technical studies	−0.121	[−0.666, 0.425]	−0.047	[−0.588, 0.495]
Education = University Degree	−0.152	[−0.692, 0.388]	−0.085	[−0.620, 0.451]
Education = Postgraduate	−0.143	[−0.684, 0.398]	−0.076	[−0.612, 0.460]
Age 2 (years)	0.001	[−0.001, 0.003]	0.001	[−0.001, 0.003]
SS x +EWB			−0.056	[−0.149, 0.037]
SS x −EWB			−0.024	[−0.114, 0.065]
BAS x +EWB			0.852 **	[0.232, 1.472]
BAS x −EWB			0.059	[−0.626, 0.746]
BAS2 x +EWB			−0.147 **	[−0.258, −0.035]
BAS2 x −EWB			−0.013	[−0.134, 0.108]
RC x +EWB			0.219	[−0.476, 0.914]
RC x −EWB			−0.858 *	[−1.569, −0.147]
RC2 x +EWB			−0.046	[−0.147, 0.055]
RC2 x −EWB			0.113 *	[0.011, 0.215]
Adjusted *R^2^*	0.379		0.419	
*F*	31.094 **		16,991 **	
Δ*R^2^*			0.026	
Δ*F*			2.148 *	

Note: Regression calculated on logarithmic transformation of anxiety symptoms scale. * *p* < 0.05; ** *p* < 0.01.

## Data Availability

Not applicable.
